# Joint Full-Duplex/Half-Duplex Transmission-Switching Scheduling and Transmission-Energy Allocation in Cognitive Radio Networks with Energy Harvesting

**DOI:** 10.3390/s18072295

**Published:** 2018-07-15

**Authors:** Tran Nhut Khai Hoan, Hiep Vu-Van, Insoo Koo

**Affiliations:** 1 School of Electrical and Computer Engineering, University of Ulsan, Ulsan 680-749, Korea; tnkhoan@ctu.edu.vn (T.N.K.H.); vvhiep@gmail.com (H.V.-V.); 2 College of Engineering Technology, Can Tho University, Can Tho City 900000, Vietnam

**Keywords:** full-duplex/half-duplex switching scheduling, energy harvesting, cognitive radio, transmission energy allocation, POMDP, actor–critic

## Abstract

The full-duplex transmission protocol has been widely investigated in the literature in order to improve radio spectrum usage efficiency. Unfortunately, due to the effect of imperfect self-interference suppression, the change in transmission power and path loss of non-line-of-sight fading channels will strongly affect performance of full-duplex transmission mode. This entails that the full-duplex transmission protocol is not always a better selection compared to the traditional half-duplex transmission protocol. Considering solar energy-harvesting-powered cognitive radio networks (CRNs), we investigate a joint full-duplex/half-duplex transmission switching scheduling and transmission power allocation in which we utilize the advantages of both half-duplex and full-duplex transmission modes for maximizing the long-term throughput of cognitive radio networks. First, we formulate the transmission rate of half-duplex and full-duplex links for fading channels between cognitive user and base station in which the channel gain is assumed to follow an exponential distribution. Afterward, by considering the availability probability of the primary channel, the limitation of the energy-harvesting capacity of the cognitive user, and the transmission capacity of half-duplex and full-duplex links, we describe the problem in terms of long-term expected throughput. The problem is then solved by adopting the partially observable Markov decision process framework to find the optimal transmission policy for the transmission pair between cognitive user and base station in order to maximize the long-term expected throughput. The optimal policy consists of either the half-duplex or the full-duplex transmission protocols as well as the corresponding amount of transmission energy in each time slot. In addition, to reduce the complexity in formulation and calculation, we also apply the actor–critic-based learning method to solve the considered problem. Finally, the performance of the proposed scheme was evaluated by comparing it with a conventional scheme in which the context of energy harvesting and long-term throughput is not considered.

## 1. Introduction

The family of conventional half-duplex (HD) wireless communication systems relied on transmitting and receiving in different time slots or frequency sub-bands, which leads to an erosion of resource utilization. Due to the demand of delivering higher data rates in wireless communications under the scarcity of radio resources, the spectrum usage efficiency has to be further improved. Thus, wireless research community aspires to conceive full-duplex (FD) communications for supporting concurrent transmission and reception in a single time/frequency channel, which is expected to improve the attainable spectral usage efficiency by a factor of two compared to HD communications [1,2,3]. However, one of the key challenges for FD transmission is to mitigate the strong effect of self-interference on the signal-to-noise ratio (SNR) at both receivers. Therefore, there have been numerous studies in the literature that focus on the development of the self-interference cancellation technique, as demonstrated by Zhang et al. [1], Kim et al. [2], Alves et al. [3], and the references therein. Many researchers have already demonstrated the feasibility of FD communication in practical systems [4,5,6,7,8].

Practically, we can not completely suppress the effect of self-interference due to the hardware imperfections [1,2,3,4,5,9,10]; hence, performance of FD communications directly depends on the self-interference suppression factor, the channel gain between communications devices, and the transmission power at both transmitters. When operating in non-line-of-sight (non-LOS) fading channels, the FD transmission protocol may outperform its HD counterpart when the channel gain between communications devices becomes high. However, when the channel gain worsens, the signal received from the self-interference link will dominate the received signal of interest and critically affect the FD link capacity. In such cases, the FD transmission protocol may not always the best choice, and the HD protocol should be considered. Therefore, a switching schedule between the HD protocol and the FD protocol should be developed to adaptively exploit the full advantages of both HD and FD communications according to the alternation of transmission environment.

In addition, recent observations and measurements of spectrum utilization show that a fixed radio resource allocation policy may lead to inefficient utilization of a large amount of licensed spectrum due to sporadic usage across different geographic regions as well as in different periods of time [11]. Meanwhile, the demand for wireless communications has been rapidly increasing, which opens up space for novel and efficient radio communications networks based on dynamic spectrum access, and has subsequently led to the concept of cognitive radio (CR) [12]. CR has been considered a viable solution for the problem of spectrum scarcity by allowing cognitive users (CUs) to opportunistically access the vacant licensed channels (or primary channels). Moreover, according to a recent survey, energy-harvesting-powered CR networks (CRN) have been widely studied and implemented at different levels of applications [13,14,15,16,17,18,19,20,21,22]. Despite numerous efforts to improve the energy-harvesting capacity, the harvesting rate is currently limited and greater efforts need to be made in the future [13,14,23].

In this paper, we consider an energy-harvesting-based CRN in which energy budget for use by CUs is very limited due to the constraint on low rate of energy-harvesting. To improve the overall transmission rate of the CRN, we additionally consider the capability of using FD links between CU and base station (BS). In FD operation, when transmission power from both devices is low, FD mode may provide a higher transmission rate compared to its HD counterpart due to the weak impact of self-interference. However, when transmission power increases, since self-interference becomes stronger, FD mode is possibly no longer a better protocol compared to HD communications. Generally, when channel gain between both transmitting devices and self-interference suppression factor are given, we can choose either HD or FD transmission modes, which can provide better performance according to the transmission power of both transmitters. However, in the scenario of energy-harvesting-based CRNs, in a time slot, when we maximize the transmission power (or transmission energy) by both BS and CU in a time slot and choose an appropriate transmission protocol, we may achieve higher transmission rates in that current time slot. Due to the low harvesting rate, CUs may lack energy for use in the next time slots and may not efficiently utilize the primary channels; hence, the average throughput (defined as the average transmission rate of the CU–BS link during the whole operation time of that link) will decrease. Therefore, decision on the amount of transmission energy in the current time slot not only affects the immediate transmission rate in that time slot but also the future reward in the next time slots. This paper investigates a joint FD/HD transmission mode switching schedule and transmission energy allocation in order to maximize long-term average throughput of energy-harvesting-powered CRN.

### 1.1. Main Contributions

The key contributions of this paper include as follows:

- We propose a scheme to find the joint optimization of an FD/HD mode switching schedule and transmission energy allocation corresponding to each transmission protocol. We analyze and formulate the expressions of the transmission rate of FD and HD links for non-LOS fading channel between CU and BS in which the channel gain is assumed to follow an exponential distribution. The target of this paper is to improve the long-term average throughput for a CU–BS transmission pair in energy-harvesting-powered CRNs.
- Considering the availability of the potential primary channel, the limitation rate of energy-harvesting of CU and the transmission capacity of HD and FD links, we describe the problem in terms of long-term expected throughput. We then formulate and solve the problem by adopting the partially observable Markov decision process (POMDP) framework to find the optimal transmission policy for CU–BS transmission pair in order to maximize the long-term expected throughput. In each time slot, the optimal policy consists of either the HD or FD transmission protocol as well as the corresponding amount of transmission energy.
- To reduce the complexity in formulation and calculation of the POMDP method, we also apply the actor–critic-based learning method to solve the considered problem in which the optimal transmission policy can be obtained directly during the learning process.
- The simulation revealed that the average throughput of the proposed scheme solved by both POMDP and actor–critic methods greatly improved compared to that of the conventional scheme [6] in which the context of energy harvesting and long-term throughput were not considered.

### 1.2. Related Work

Lately, there have been significant studies on FD communications; herein, we briefly summarize the most relevant papers to this work. Compared to our proposed scheme, the most related works in the literature focus on investigating the performance of wireless FD communications compared to HD counterparts as follows:

In [6], the authors compare the performance of FD and HD transmission protocol in terms of achievable throughput $\left(\mathrm{bits}/s/\mathrm{Hz}\right)$ according to self-interference factor for CRNs. Thereby, the authors show that, when self-interference factor changes, there exists a crossing-point where we should switch between HD and FD modes to attain more throughput.

In [7], considering the imperfect self-interference cancellation and some hardware constraints, the authors compare wireless HD and FD communication in three communication scenarios: two-way channel, two-hop (relaying) channel, and a two-way two-hop (two-way relaying) systems. Their analysis provided the guidelines for the selection of an HD or FD mode for the considered scenarios. The decision of either an HD mode or FD mode is based on the degrees of freedom (DoFs) analysis, which characterizes the achievable rate corresponding to each each communication mode.

In [8], the authors propose a transmission mode selection scheme device-to-device (D2D) communications underlay cellular networks. They investigated the performance for hybrid FD/HD-D2D communications and present a tractable analytical framework for a hybrid-duplex D2D-enabled cellular network. Each pair of communication devices is considered to perform in FD-D2D mode, perform in HD-D2D mode, or stay silent according to the caused interference from the potential D2D pair to the BS and the distance between two devices. The target of [8] is spectrum efficiency, which is expressed in terms of normalized achievable rate per square unit $\left(\mathrm{nats}/s/\mathrm{Hz}/km^{2}\right)$.

In [17], the authors investigate the optimal cognitive sensing and access policies for a secondary user in energy harvesting CRNs. Focusing on secondary throughput, the authors formulated and solved the problem based on the POMDP framework, and subsequently investigated the variation of throughput with various system parameters. However, the FD transmission mode and non-LOS fading channel were not considered in this work.

In [21], the authors propose a resource allocation scheme for unmanned aerial vehicle (UAV)-assisted networks in which an UAV acting as an energy source provides radio energy for multiple energy-harvesting-powered D2D transmission pairs with much information to be transmitted. The goal is to maximize the average throughput of UAV-assisted networks. Subsequently, in [22], the authors present a vision of UAV-supported ultra dense networks (UDNs), show the broad applications of UAV-supported UDNs in communications, caching, and energy transfer, and highlight the efficient power control in UAV-supported UDNs. In addition, the authors also discuss the dominating technical challenges and open issues ahead.

In short, works in the literature have paid great attention to energy-harvesting-powered networks and applications. Generally, the aforementioned works already compared the throughput of the HD protocol and the FD protocol, which allows switching between these transmission modes to mitigate the drawback of each transmission protocol according to the variation in transmission environment, e.g., imperfect self-interference suppression and the channel gain between the two transmission devices. However, these schemes do not consider energy-harvesting scenarios in which the energy budget for use by transmitting devices is limited. Therefore, the aforementioned schemes will utilize the greedy algorithm in which transmitting devices try to maximize transmission power and to decide its corresponding transmission protocol (either HD or FD) in order to maximize the immediate achievable rate only for the current transmission without considering any future reward. When operating in energy-harvesting scenarios, transmission devices may lack energy for use in the next transmissions and have to stay silent; hence, overall throughput will decrease. In the proposed scheme, we investigate the joint optimization of the FD/HD mode switching schedule and the transmission energy allocation corresponding to each transmission protocol.

The rest of the paper is organized as follows. Section 2 details the model of the considered network and assumptions. In Section 3, we formulate the expressions for calculating the expected transmission rates of HD and FD transmission links. The formulation for the proposed scheme by adopting the POMDP framework and solution are shown in Section 4. Our solution by actor–critic-based learning method is presented in Section 5. Simulation results and discussion are detailed in Section 6. The work is concluded in Section 7.

## 2. Network Model and Assumptions

**Figure 1:**
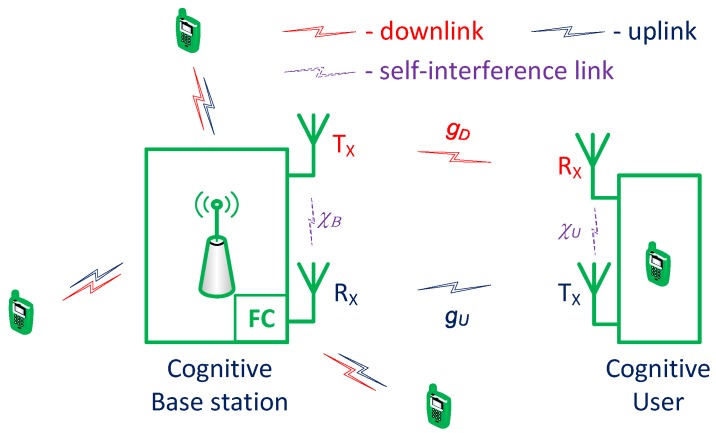
Model of the considered system.

Herein, we consider a BS–CU transmission pair in centralized CRNs in which a number of CUs, centered by a BS, utilize a number of potential time-slotted primary channels, as shown in Figure 1. The BS is integrated with a fusion center (FC) used for making a global decision based on local sensing results sent from CUs. In addition, each CU is equipped with two separate antennas: one for transmitting and the other for receiving. Hence, in each time slot, the communications link between a CU and the BS can be configured either to transmit and receive simultaneously (FD mode), or to transmit and receive in turn in each half of the time slot (HD mode). Figure 1 shows the model of the considered system, where $\chi_{U}$ and $\chi_{B}$ denote the self-interference factors at the CU and BS, respectively, when the communications link is in FD mode.

The energy for use at each CU hails from solar-energy-harvesting. Herein, we consider a practical scenario in which the amount of harvested energy is very limited [13]. In this work, the energy harvesting model can be discretely approximated as follows. Let $E_{hv}$ be the amount of harvested energy (packets) during a time slot, which is stored in a rechargeable battery and can be used for next time slots. Let $E_{Bat}$ be the battery capacity of the CU. The amount of harvested energy, $E_{hv}$, in each interval of time slot can be approximated as a Poisson process with mean value $E_{hvmean}$ [24,25]. Thus, $E_{hv}$ can be expressed as $E_{hv}=\varepsilon_{hv}\left(k\right)$ where k=0,1,2,... and $0\leq \varepsilon_{hv}\left(0\right)<\varepsilon_{hv}\left(1\right)<\varepsilon_{hv}\left(2\right),...,<E_{Bat}$. Subsequently, the probability mass function of $E_{hv}$ can be approximated as [25]

(1) $$p_{hv}\left(k\right)=\frac{{\left(E_{hvmean}\right)}^{k}}{k!}e^{-E_{hvmean}},k=0,1,2,...$$

**Figure 2:**
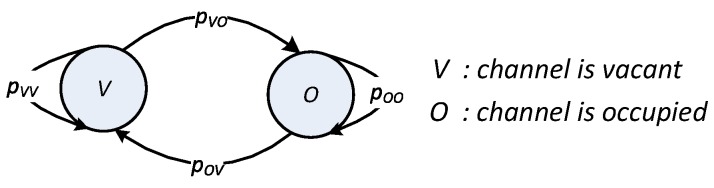
Model of the primary channel.

In each time slot, the state of each primary channel is represented as $H\in \left\{V,O\right\}$, where *V* and *O* denote the hypotheses that the channel is vacant or occupied, respectively. The state transition between two adjacent time slots is modeled as a two-state discrete-time Markov chain process, as shown in Figure 2, where $p_{ij}:i,j\in \left\{V,O\right\}$ denotes the transition probability from state *i* in the current time slot to state *j* in the next time slot. How these transition probabilities are obtained has been well-studied in the literature [26,27,28,29]; hence, we assume that the transition probabilities are known a priori. These transition probabilities are used to update the probability that the channel is vacant in the next time slot according to the available probability of the channel in the current time slot (also called the belief). Let μ(t) be the belief of the primary channel in current time slot *t*. The belief in the next time slot (t+1) can be calculated as follows:

(2) $$\mu \left(t+1\right)=\mu \left(t\right)p_{VV}+\left(1-\mu (t)\right)p_{OV}.$$

**Figure 3:**
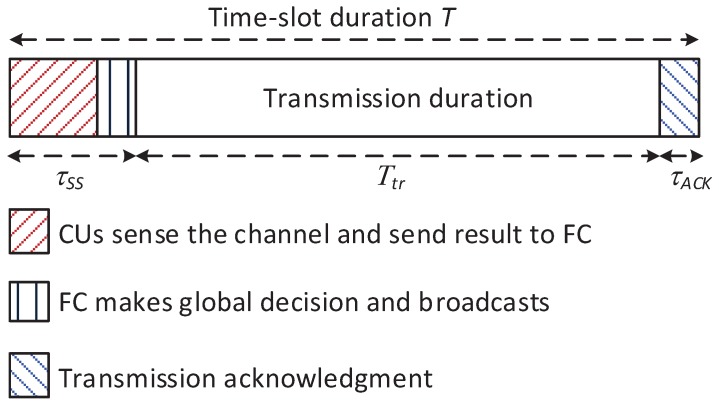
Time frame for the operation of a cognitive radio network (CRN) in a time slot.

Additionally, let us consider that the link for each CU–BS transmission pair is assigned to one specific primary channel and the duration in which this channel is assigned to the transmission pair is much longer than one time slot. Thus, scheduling the long-term operation of this transmission pair over multiple time slots is considered in this work. Figure 3 illustrates the time frame for the operation of the CRN in one time slot. At the beginning of each time slot, to improve the reliability of the sensing process, collaborative spectrum sensing (CSS) is performed to detect a set of potential primary channels. After the CSS process, based on sensing reports from CUs, the FC makes a global decision for these channels and broadcasts it on a private common control channel. Ways of guaranteeing a common control channel in CRNs have been well-studied in other literature; thus, we assume that the common control channel is available here. According to the global decision, each CU–BS transmission pair decides either to stay silent or to start its transmission process on the channel assigned to them during the remaining time of the current time slot. Since CSS has been well studied in the literature [30,31] and the proposed algorithm is not dependent on combination rules at the FC, this work does not investigate CSS; we assume that the global probability of false alarm, $p_{f}$, and the global probability of detection, $p_{d}$, are given. Hereafter, we focus on improving the performance of a CU–BS transmission pair on the primary channel assigned to it.

We consider non-line-of-sight (NLOS) fading channel such that the channel gain of both links varies identically and independently across time slots; however, we assume that the channel gain remains constant during an entire time slot [32]. Let $g_{U}$ and $g_{D}$ be the channel power gain of uplink and downlink, respectively. The variation of $g_{U}$ and $g_{D}$ is assumed to follow exponential distribution with the mean values of $G_{U}$ and $G_{D}$, respectively [33,34]. We do not focus on investigating the correlation between uplink and downlink; thus, we model uplink and downlink as the NLOS fading channel in a general case without considering the reciprocal relation between them. The assumption of uplink and downlink, moreover, does not have any effect on the operation the proposed algorithm. Furthermore, the proposed scheme can also be used with any other model of uplink and downlink when the reciprocal relation between them is considered. We will detail the formulation of FD link and HD link in the next section.

## 3. Formulation of HD and FD Links

### 3.1. FD Link

In each time slot, after the sensing phase, when the global decision indicates that the primary channel is vacant, the BS and CU can start their transmission process. Let $G_{0U}$ and $G_{0D}$ be the measurement values of channel gain for the uplink and downlink, respectively, in the current time slot. We assume that $G_{0D}$ and $G_{0U}$ in the current time slot are available. In fact, these can be measured periodically or from the previous transmission. The measurement methods are well studied in the literature, i.e. the transmitter can send known channel-estimation pilots to the receiver, where the channel gain can be estimated according to these pilots and the background noise at the receiver [35,36]. The expected transmission rate (nats/s/Hz) achieved when FD transmission mode is used can be given as follows:

(3) $$\begin{array}{c} R_{FD}=\frac{T_{tr}}{T}\left\{\underset{\left(1\right)}{\underset{︸}{\ln\left(1+\frac{\frac{G_{0D}E_{trD}}{T_{tr}}}{1+\frac{\chi_{U}E_{trU}}{T_{tr}}}\right)}}\right.+\left.\underset{\left(2\right)}{\underset{︸}{\ln\left(1+\frac{\frac{G_{0U}E_{trU}}{T_{tr}}}{1+\frac{\chi_{B}E_{trD}}{T_{tr}}}\right)}}\right\} \end{array}$$

where $T_{tr}$ denotes the transmitting duration; $E_{trU}$ and $E_{trD}$ denote the amount of transmission energy used by the CU and BS, respectively, $0\leq E_{trU},E_{trD}\leq E_{trMAX}$. Equation (3) consists of two terms: Term (1) denotes the expected transmission rate on the downlink, which is transmitted by the BS and received by the CU; Term (2) denotes the rate on the uplink, which is transmitted by the CU and received by the BS. We note that $R_{FD}$ denotes the expected transmission rate when transmission is successful, and the CU and the BS may receive data at different rates. This is because the required transmission rates on uplink and downlink are generally asymmetric; in addition, the channel gain of uplink and downlink may also be different in non-LOS fading channel. Due to the fact that the energy-harvesting rate is very low, the amount of energy for use by the CU is assumed to be limited. Although the energy budget for use at by the BS is not limited, the transmission energy by BS needs to be controlled since maximizing transmission energy by the BS will seriously increase interference to the uplink at its own receiver. Thus, we need to decide beforehand the transmission energy, $E_{trU}$, at the CU; we then find the transmission energy, $E_{trD}$, at the BS such that the following condition should be satisfied:

(4) $$\begin{array}{c} \underset{\left(1\right)}{\underset{︸}{\frac{\frac{G_{0D}E_{trD}}{T_{tr}}}{1+\frac{\chi_{U}E_{trU}}{T_{tr}}}}}=\eta \underset{\left(2\right)}{\underset{︸}{\frac{\frac{G_{0U}E_{trU}}{T_{tr}}}{1+\frac{\chi_{B}E_{trD}}{T_{tr}}}}} \end{array}$$

where the terms (1) and (2) denote the required signal-to-interference-plus-noise ratio (SINR) at the BS and CU receivers, respectively, and η denotes the asymmetric coefficient on the uplink compared to the downlink, which is set based on the demand on capacity of the uplink and downlink. Solving Equation (4) yields $E_{trD}$ as given in Equation (5). Although there is no energy constraint at the BS, in this work, $E_{trD}$ is practically limited by a specific maximum transmission energy, $E_{trDMAX}$.

(5) $$\begin{array}{c} E_{trD}=\frac{-T_{tr}+\sqrt{{T_{tr}}^{2}+4\eta \chi_{B}\frac{G_{0U}}{G_{0D}}\left(\chi_{U}{E_{trU}}^{2}+T_{tr}E_{trU}\right)}}{2\chi_{B}}. \end{array}$$

We use Equation (3) to calculate transmission rate of FD link when the channel gain on uplink and downlink are given in the current time slot.

In any time slot, when the channel gain on uplink and downlink are not given, we can estimate the expected transmission rate in that slot based on the exponential probability density functions of $g_{U}$ and $g_{D}$ as follows:

(6) R˜FD=TtrT∫0∞ln1+gUEtrUTtr1+χBEtrDTtr1GUe-gUGUdgU+∫0∞ln1+gDEtrDTtr1+χUEtrUTtr1GDe-gDGDdgD

which can be rewritten as

(7) $$\begin{array}{c} {\tilde{R}}_{FD}=\frac{T_{tr}}{T}\left\{\int_{0}^{\infty }\ln\left(1+g_{FDU}\right)\frac{e^{\frac{-g_{FDU}}{G_{FDU}}}}{G_{FDU}}d\left(g_{FDU}\right)\right.+\left.\int_{0}^{\infty }\ln\left(1+g_{FDD}\right)\frac{e^{\frac{-g_{FDD}}{G_{FDD}}}}{G_{FDD}}d\left(g_{FDD}\right)\right\} \end{array}$$

where $g_{FDU}=g_{U}\frac{\frac{E_{trU}}{T_{tr}}}{1+\frac{\chi_{B}E_{trD}}{T_{tr}}}$, $g_{FDD}=g_{D}\frac{\frac{E_{trD}}{T_{tr}}}{1+\frac{\chi_{U}E_{trU}}{T_{tr}}}$, $G_{FDU}=G_{U}\frac{\frac{E_{trU}}{T_{tr}}}{1+\frac{\chi_{B}E_{trD}}{T_{tr}}}$, and $G_{FDD}=G_{D}\frac{\frac{E_{trD}}{T_{tr}}}{1+\frac{\chi_{U}E_{trU}}{T_{tr}}}$. After calculating the integrals in Equation (7) and doing some mathematical transformations, ${\tilde{R}}_{FD}$ can be expressed in the following form:

(8) $$\begin{array}{c} {\tilde{R}}_{FD}=\frac{T_{tr}}{T}\left\{e^{\frac{1}{G_{FDU}}}\int_{\frac{1}{G_{FDU}}}^{\infty }\frac{e^{-t}}{t}dt\right.+\left.e^{\frac{1}{G_{FDD}}}\int_{\frac{1}{G_{FDD}}}^{\infty }\frac{e^{-t}}{t}dt\right\} \end{array}$$

where $G_{FDU}$ and $G_{FDD}$ denote the average value of the SINR at the BS and CU receivers, respectively. Here, we also need to find the expression of $E_{trD}$ at the BS that satisfies the condition $G_{FDU}=\eta \times G_{FDD}$, which can be expressed in Equation (9) as follows:

(9) $$\begin{array}{c} G_{D}\frac{\frac{E_{trD}}{T_{tr}}}{1+\frac{\chi_{U}E_{trU}}{T_{tr}}}=\eta G_{U}\frac{\frac{E_{trU}}{T_{tr}}}{1+\frac{\chi_{B}E_{trD}}{T_{tr}}}. \end{array}$$

Solving Equation (9) yields $E_{trD}$ as expressed in Equation (10):

(10) $$\begin{array}{c} E_{trD}=\frac{-T_{tr}+\sqrt{{\left(T_{tr}\right)}^{2}+4\eta \chi_{B}\frac{G_{U}}{G_{D}}\left(\chi_{U}{\left(E_{trU}\right)}^{2}+T_{tr}E_{trU}\right)}}{2\chi_{B}}. \end{array}$$

### 3.2. HD Link

In the HD link, transmission duration in each time slot is divided into two sub-slots. Although the transmission duration on the uplink and downlink may be different, two sub-slots are equally divided for avoiding complicated variable definitions here. The first sub-slot is used for transmission of the downlink and the second one is used for the uplink. Thus, there is no interference between uplink and downlink transmissions. Similar to the FD link, when channel gains $G_{0U}$ and $G_{0D}$ on the uplink and downlink, respectively, are given, the expected transmission rate (nats/s/Hz), achieved when HD transmission mode is used, can be obtained as follows:

(11) $$\begin{array}{c} R_{HD}=\frac{1}{2}\frac{T_{tr}}{T}\left(\ln\left(1+\frac{G_{0U}E_{trU}}{T_{tr}/2}\right)+\ln\left(1+\frac{G_{0D}E_{trD}}{T_{tr}/2}\right)\right). \end{array}$$

We also need to find transmission energy $E_{trD}$ according to $E_{trU}$ as follows:

(12) $$\begin{array}{c} \frac{G_{0D}E_{trD}}{T_{tr}}=\beta \frac{G_{0U}E_{trU}}{T_{tr}}\Rightarrow E_{trD}=\eta \frac{G_{0U}}{G_{0D}}E_{trU}. \end{array}$$

In any time slot, when the channel gain on the uplink and downlink are not given, we can also estimate the expected transmission rate in that time slot based on the exponential probability density functions of $g_{U}$ and $g_{D}$ as follows:

(13) $$\begin{array}{c} {\tilde{R}}_{HD}=\frac{1}{2}\frac{T_{tr}}{T}\left\{\int_{0}^{\infty }\ln\left(1+\frac{g_{U}E_{trU}}{T_{tr}/2}\right)\frac{1}{G_{U}}e^{\frac{-g_{U}}{G_{U}}}dg_{U}\right.+\left.\int_{0}^{\infty }\ln\left(1+\frac{g_{D}E_{trD}}{T_{tr}/2}\right)\frac{1}{G_{D}}e^{\frac{-g_{D}}{G_{D}}}dg_{D}\right\} \end{array}$$

which can also be transformed into the following expression:

(14) $$\begin{array}{c} {\tilde{R}}_{HD}=\frac{T_{tr}}{2T}\left\{\int_{0}^{\infty }\ln\left(1+g_{HDU}\right)\frac{e^{\frac{-g_{HDU}}{G_{HDU}}}}{G_{HDU}}d\left(g_{HDU}\right)\right.+\left.\int_{0}^{\infty }\ln\left(1+g_{HDD}\right)\frac{e^{\frac{-g_{HDD}}{G_{HDD}}}}{G_{HDD}}d\left(g_{HDD}\right)\right\} \end{array}$$

where $g_{HDU}=\frac{g_{U}E_{trU}}{T_{tr}/2}$, $g_{HDD}=\frac{g_{D}E_{trD}}{T_{tr}/2}$, $G_{HDU}=\frac{G_{U}E_{trU}}{T_{tr}/2}$, and $G_{HDD}=\frac{G_{D}E_{trD}}{T_{tr}/2}$. Calculating the integrals in Equation (14) and doing some mathematical transformations yield the expression of ${\tilde{R}}_{HD}$ as follows:

(15) $$\begin{array}{c} {\tilde{R}}_{HD}=\frac{T_{tr}}{2T}\left\{e^{\frac{1}{G_{HDU}}}\int_{\frac{1}{G_{HDU}}}^{\infty }\frac{e^{-t}}{t}dt\right.+\left.e^{\frac{1}{G_{HDD}}}\int_{\frac{1}{G_{HDD}}}^{\infty }\frac{e^{-t}}{t}dt\right\} \end{array}$$

where $G_{HDU}$ and $G_{HDD}$ denote the average SINR at the BS and CU receivers, respectively. Similarly, we also find the expression of $E_{trD}$ at the BS according to $E_{trU}$ as follows:

(16) $$\begin{array}{c} \frac{G_{D}E_{trD}}{T_{tr}/2}=\eta \frac{G_{U}E_{trU}}{T_{tr}/2}\Rightarrow E_{trD}=\eta \frac{G_{U}}{G_{D}}E_{trU}. \end{array}$$

## 4. POMDP-Based HD/FD Transmission Protocol Switching Scheduling

Practically, since the operation duration of a system is much longer than the duration of a time slot, the target of this work is the long-term reward. Due to the limitation in energy-harvesting capacity during a time slot, a decision on how much energy is used for the transmission of the CU in the current time slot not only affects the immediate transmission rate of that time slot but also affects the energy budget for use by CU in the next time slots and, subsequently, affects the future reward. In addition, the transmission rate of the FD link heavily depends on the transmission energy of its transmitters. When transmission energy increases, the transmission rate of the FD link increases very slowly, compared to that of the HD link, due to the stronger effect of self-interference on its own receiver. Thus, in each time slot, based on the amount of transmission energy, we can choose either the FD or the HD transmission protocol to achieve a higher transmission rate. This section details the joint HD/FD transmission switching schedule and transmission energy allocation by adopting the POMDP framework in order to improve the long-term average transmission rate of a BS–CU transmission pair.

**Figure 4:**
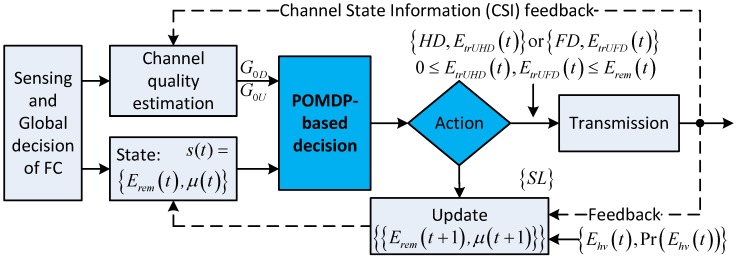
Flowchart of the proposed scheme when adopting the partially observable Markov decision process (POMDP) framework.

Figure 4 illustrates the solution based on the POMDP framework. In the time slot $t_{0}$, after the CSS phase, when the global decision indicates that the channel is occupied, the CU trusts this result and stays silent during the remaining duration of the time slot. At the end of the time slot, the CU updates the amount of harvested energy during the time slot, the channel state probability (also called ***belief***), and the remaining energy for use in the next time slots. Note that, for simplicity, this case is not shown in Figure 4. On the other hand, when the channel is vacant, based on the energy remaining in the battery, $E_{rem}\left(t_{0}\right)$, the belief for the primary channel, $\mu \left(t_{0}\right)$ and the channel state information (CSI) on about channel gain between the CU and the BS, the CU and the BS will choose the optimal action from among $\left\{HD,E_{trUHD}\left(t_{0}\right)\right\}$ for the HD with transmission energy $E_{trUHD}\left(t_{0}\right)$, $\left\{FD,E_{trUFD}\left(t_{0}\right)\right\}$ for the FD with transmission energy $E_{trUFD}\left(t_{0}\right)$, and staying in silent mode. The optimal action in time slot $t_{0}$ depends on the summation of the immediate reward in the current time slot $t_{0}$ and the expected future reward from time slot $t=t_{0}+1$. The expected future reward is formulated based on the POMDP framework as follows.

*State space S:* In time slot *t*, the CU decides the action based on the remaining energy in the battery $E_{rem}\left(t\right)$ and the belief about the availability of the primary channel $\mu \left(t\right)$. Hence, each state $s\left(t\right)\in S$ is defined as $s\left(t\right)=\left\{E_{rem}\left(t\right),\mu \left(t\right)\right\}$.

*Action space A:* In time slot *t*, the CU decides on action $a\left(t\right)$ which is one of the operation modes in action space *A* defined as $A=\left\{\left\{SL\right\},\left\{HD,E_{trUHD}\left(t\right)\right\},\left\{FD,E_{trUFD}\left(t\right)\right\}\right\}$, which consists of staying in silent mode, $\left\{SL\right\}$, transmitting by using HD transmission mode, $\left\{HD,E_{trUHD}\left(t\right)\right\}$, and transmitting by using FD transmission mode, $\left\{FD,E_{trUFD}\left(t\right)\right\}$, where $E_{trUHD}\left(t\right)$ and $E_{trUFD}\left(t\right)$ denote the optimal amount of the transmission energy of the corresponding HD or FD transmission modes, respectively.

*Reward:* Given state $s\left(t\right)=\left\{E_{rem}\left(t\right),\mu \left(t\right)\right\}$, each action $a\left(t\right)\in A$ is accompanied by a corresponding reward, $R_{W}\left(s\left(t\right),a\left(t\right)\right)$. The reward is defined as the expected transmission rate in the time slot when transmission is successful (when Acknowledge [ACK] is received at the end of the transmission phase); otherwise, the reward is zero when no ACK or negative ACK (NACK) is received. In addition, zero throughput is the penalty when action $a\left(t\right)$ is SL.

### 4.1. Silent Mode $\left(\Theta_{1}\right)$

In time slot *t*, when the global decision indicates that the primary channel is occupied, the CU will trust this result and stay in silent mode. In this case, no throughput is attained, such that $R_{W}\left(E_{rem}\left(t\right),\mu \left(t\right),a\left(t\right)\left|\Theta_{1}\right.\right)=0$. The probability that this action occurs is calculated as $\mathrm{Pr}\left[\Theta_{1}\right]=\underset{\left(1\right)}{\underset{︸}{\mu \left(t\right)p_{f}}}+\underset{\left(2\right)}{\underset{︸}{\left(1-\mu \left(t\right)\right)p_{d}}}$, where Term (1) denotes the probability that the channel is detected as occupied but is actually vacant, whereas Term (2) denotes the probability that the channel is correctly detected as vacant; $p_{d}$ and $p_{f}$ denote the probability of detection and false alarm, respectively, of the sensing scheme. At the end of time slot *t*, belief $\mu \left(t+1\right)$ for the next time slot, t+1, can be updated according to Bayes’ rule and state transition probabilities (shown in Figure 2) as

(17) $$\begin{array}{c} \mu \left(t+1\right)=\frac{\mu \left(t\right)p_{f}}{\mathrm{Pr}\left[\Theta_{1}\right]}p_{VV}+\frac{\left(1-\mu \left(t\right)\right)p_{d}}{\mathrm{Pr}\left[\Theta_{1}\right]}p_{OV} \end{array}$$

where $p_{VV}$ and $p_{OV}$ denote the transition probabilities given in Figure 2. The remaining energy, $E_{rem}\left(t+1\right)$, for use in the next time slot, t+1, can be updated as

(18) $$\begin{array}{c} E_{rem}\left(t+1\right)=\min\left\{E_{Bat},E_{rem}\left(t\right)-E_{SS}+E_{hv}\left(t\right)\right\} \end{array}$$

where $E_{SS}$ stands for the energy consumed during the sensing phase in one time slot. The transition probability of energy from current time slot *t* to the next time slot, t+1, can be expressed as Equation (19), where $p_{hv}\left(k\right)$ is given in Equation (1).

(19) $$\begin{array}{ccc} \mathrm{Pr}\left[E_{rem}\left(t\right)\to E_{rem}\left(t+1\right)\right] & = & \mathrm{Pr}\left[E_{hv}\left(t\right)=\varepsilon_{hv}\left(k\right)\right]\\ & = & p_{hv}\left(k\right). \end{array}$$

### 4.2. HD Transmission Mode

In time slot *t*, when the global decision indicates that the primary channel is vacant. The probability that this event occurs is $\underset{\left(1\right)}{\underset{︸}{\mu \left(t\right)\left(1-p_{f}\right)}}+\underset{\left(2\right)}{\underset{︸}{\left(1-\mu \left(t\right)\right)\left(1-p_{d}\right)}}$, where Term (1) denotes the probability that primary channel is correctly detected as vacant, and Term (2) denotes the probability that the primary channel is detected as vacant while it is occupied. In this case, when the CU decides to transmit in HD mode, action $a\left(t\right)$ is decided as $\left\{HD,E_{trUHD}\left(t\right)\right\}$, where $0\leq E_{trUHD}\left(t\right)\leq E_{trMAX}$. The reward is achieved according to the observation at the end of the transmission phase. There are two possible observations which are detailed as follows.

#### 4.2.1. Observation 1 $\left(\Theta_{2}\right)$

Transmission is successful when the ACK is signaled at the end of the transmission phase. The probability that this event occurs can be calculated as

(20) $$\begin{array}{c} \mathrm{Pr}\left[\Theta_{2}\right]=\mu \left(t\right)\left(1-p_{f}\right). \end{array}$$

In this case, we assume that the primary channel is vacant (state V) during time slot *t*; hence, belief $\mu \left(t+1\right)$ for the next time slot, t+1, can be updated as

(21) $$\begin{array}{c} \mu \left(t+1\right)=p_{VV}. \end{array}$$

The remaining energy, $E_{rem}\left(t+1\right)$, for use in the next time slot, t+1, can be updated as

(22) $$\begin{array}{c} \begin{array}{c} E_{rem}\left(t+1\right)=\min\left\{E_{Bat},E_{rem}\left(t\right)-E_{trUHD}\left(t\right)-E_{recHD}-E_{SS}+E_{hv}\left(t\right)\right\} \end{array} \end{array}$$

when $E_{rem}\left(t\right)-E_{trUHD}\left(t\right)-E_{recHD}-E_{SS}\geq 0$; otherwise,

(23) $$\begin{array}{c} E_{rem}\left(t+1\right)=\min\left\{E_{Bat},E_{rem}\left(t\right)-E_{SS}+E_{hv}\left(t\right)\right\} \end{array}$$

where $E_{recHD}$ denotes the energy spent during the HD receiving process in one time slot which is the same for all time slots. The transition probability of energy from current time slot *t* to the next time slot, t+1, is also given in Equation (19). Equation (23) denotes the case when the remaining energy, $E_{rem}\left(t\right)$, is not sufficient for transmission when the amount of transmission energy is $E_{trUHD}\left(t\right)$. The reward achieved in this case can be formulated as follows. When the remaining energy is not sufficient such that $E_{rem}\left(t\right)-E_{trUHD}\left(t\right)-E_{recHD}-E_{SS}<0$, no transmission can be decided, and the reward is

(24) $$\begin{array}{c} R_{W}\left(E_{rem}\left(t\right),\mu \left(t\right),a\left(t\right)=\left\{HD,E_{trUHD}\left(t\right)\right\}\left|\Theta_{2}\right.\right)=0. \end{array}$$

Otherwise, the reward should be designated by Equation (15) as ${\tilde{R}}_{HD}\left(E_{trUHD}\left(t\right)\right)$. Since the battery capacity is finite, if the CU stays in silent mode for a long time, the battery will be full. When the amount of harvested energy during a time slot becomes more than the storable space of the battery, the battery will overflow and the residual harvested energy will be wasted. Let us name this overflow event as $OF_{HD}$, which can be expressed as follows:

(25) $$OF_{HD}=\left\{\begin{array}{c} \begin{array}{cc} 1 & \mathrm{if}\left(\begin{array}{c} E_{rem}\left(t\right)-E_{trUHD}\left(t\right)-E_{recHD}-E_{SS}+E_{hv}\left(t\right) \end{array}\right)>E_{Bat} \end{array}\\ \begin{array}{cc} 0 & \mathrm{otherwise} \end{array} \end{array}\right..$$

To avoid entering the overflow state as designated in Equation (25), we define a penalty factor $\xi_{HD}$ as

(26) $$\begin{array}{c} \xi_{HD}=\left\{\begin{array}{c} \begin{array}{cc} 0, & OF_{HD}\&\left(E_{trUHD}\left(t\right)\neq E_{trMAX}\right) \end{array}\\ \begin{array}{cc} 1, & \mathrm{otherwise} \end{array} \end{array}\right.. \end{array}$$

Subsequently, the reward can be given as

(27) $$\begin{array}{c} R_{W}\left(E_{rem}\left(t\right),\mu \left(t\right),a\left(t\right)\left|\Theta_{2}\right.\right)=\xi_{HD}{\tilde{R}}_{HD}\left(E_{trUHD}\left(t\right)\right) \end{array}$$

where ${\tilde{R}}_{HD}\left(E_{trUHD}\left(t\right)\right)$ is given in Equation (15). Equation (27) guarantees that the maximum transmission energy will be used when the battery probably overflows at the end of the time slot.

#### 4.2.2. Observation 2 $\left(\Theta_{3}\right)$

Transmission is unsuccessful when no ACK or NACK is signaled at the end of the transmission phase. The probability that this event occurs is given as

(28) $$\begin{array}{c} \mathrm{Pr}\left[\Theta_{3}\right]=\left(1-\mu \left(t\right)\right)\left(1-p_{d}\right). \end{array}$$

In this case, we assume that mis-detection occurred and the channel is occupied (state O) during the time slot; hence, belief $\mu \left(t+1\right)$ for the next time slot, t+1, can be updated as

(29) $$\begin{array}{c} \mu \left(t+1\right)=p_{OV}, \end{array}$$

and the reward is given as

(30) $$\begin{array}{c} R_{W}\left(E_{rem}\left(t\right),\mu \left(t\right),a\left(t\right)\left|\Theta_{3}\right.\right)=0. \end{array}$$

Transition probability $\mathrm{Pr}\left[E_{rem}\left(t\right)\to E_{rem}\left(t+1\right)\right]$ is also given in (19), and the remaining energy $E_{rem}\left(t+1\right)$ for use in the next time slot, t+1, can be updated as given in (22).

### 4.3. FD Transmission Mode

In time slot *t*, this mode is also considered when the global decision indicates that the primary channel is vacant. The action is decided as $a\left(t\right)=\left\{FD,E_{trUFD}\left(t\right)\right\}$, where $0\leq E_{trUFD}\left(t\right)\leq E_{trMAX}$. Similar to HD mode, the reward is achieved based on the two possible observations at the end of the time slot, as follows.

#### 4.3.1. Observation 3 $\left(\Theta_{4}\right)$

Transmission is successful when the ACK is signaled at the end of the transmission phase. The probability that this event occurs can be calculated as

(31) $$\begin{array}{c} \mathrm{Pr}\left[\Theta_{4}\right]=\mu \left(t\right)\left(1-p_{f}\right). \end{array}$$

Similar to *Observation 1*, belief $\mu \left(t+1\right)$ for the next time slot, t+1, can be updated as given in Equation (21). Remaining energy $E_{rem}\left(t+1\right)$ for use in the next time slot can be updated as

(32) $$\begin{array}{c} \begin{array}{c} E_{rem}\left(t+1\right)=\min\left\{E_{Bat},E_{rem}\left(t\right)-E_{trUFD}\left(t\right)-E_{recFD}-E_{SS}+E_{hv}\left(t\right)\right\} \end{array} \end{array}$$

when $E_{rem}\left(t\right)-E_{trUFD}\left(t\right)-E_{recFD}-E_{SS}\geq 0$; otherwise,

(33) $$\begin{array}{c} E_{rem}\left(t+1\right)=\min\left\{E_{Bat},E_{rem}\left(t\right)-E_{SS}+E_{hv}\left(t\right)\right\} \end{array}$$

where $E_{recFD}=2\times E_{recHD}$ denotes the energy spent during the FD receiving process in one time slot. The transition probability of energy $\mathrm{Pr}\left[E_{rem}\left(t\right)\to E_{rem}\left(t+1\right)\right]$ is also given in (19). Similar to *Observation 1*, when the remaining energy is not sufficient, such that $E_{rem}\left(t\right)-E_{trUFD}\left(t\right)-E_{recFD}-E_{SS}<0$, there is no transmission, and the reward is given as

(34) $$\begin{array}{c} R_{W}\left(E_{rem}\left(t\right),\mu \left(t\right),a\left(t\right)\left|\Theta_{4}\right.\right)=0. \end{array}$$

Otherwise, when the remaining energy is sufficient and the amount of transmission energy is $E_{trUFD}\left(t\right)$, the reward is formulated as follows. Similar to the foregoing discussion of Equation (25), we also define an overflow event, $OF_{FD}$, which can be expressed as

(35) $$\begin{array}{c} OF_{FD}=\left\{\begin{array}{c} \begin{array}{cc} 1, & \mathrm{if}\left(\begin{array}{c} E_{rem}\left(t\right)-E_{trUFD}\left(t\right)-E_{recFD}-E_{SS}+E_{hv}\left(t\right) \end{array}\right)>E_{Bat} \end{array}\\ \begin{array}{cc} 0, & \mathrm{otherwise} \end{array} \end{array}\right.. \end{array}$$

The event, $OF_{FD}$, occurs when the battery overflows at the end of the time slot. Similarly, the penalty factor, $\xi_{FD}$, can be expressed based on event $OF_{FD}$ as

(36) $$\begin{array}{c} \xi_{FD}=\left\{\begin{array}{c} \begin{array}{cc} 0, & OF_{FD}\&\left(E_{trUHD}\left(t\right)\neq E_{trMAX}\right) \end{array}\\ \begin{array}{cc} 1, & \mathrm{otherwise} \end{array} \end{array}\right.. \end{array}$$

Subsequently, the reward can be given as

(37) $$\begin{array}{c} R_{W}\left(E_{rem}\left(t\right),\mu \left(t\right),a\left(t\right)\left|\Theta_{4}\right.\right)=\xi_{FD}{\tilde{R}}_{FD}\left(E_{trUFD}\left(t\right)\right) \end{array}$$

where ${\tilde{R}}_{FD}\left(E_{trUFD}\left(t\right)\right)$ is given in Equation (8). Equation (37) guarantees that the maximum transmission energy will be used when the battery probably overflows at the end of the time slot.

#### 4.3.2. Observation 4 $\left(\Theta_{5}\right)$

Transmission is unsuccessful when no ACK or NACK is signaled at the end of transmission. The probability that this event occurs is given as

(38) $$\begin{array}{c} \mathrm{Pr}\left[\Theta_{5}\right]=\left(1-\mu \left(t\right)\right)\left(1-p_{d}\right). \end{array}$$

In this case, belief $\mu \left(t+1\right)$ is updated as given in Equation (29), the transition probability of energy $\mathrm{Pr}\left[E_{rem}\left(t\right)\to E_{rem}\left(t+1\right)\right]$ is also given in Equation (19), the remaining energy, $E_{rem}\left(t+1\right)$, can be updated as given in Equation (22), and the reward is given as

(39) $$\begin{array}{c} R_{W}\left(E_{rem}\left(t\right),\mu \left(t\right),a\left(t\right)\left|\Theta_{5}\right.\right)=0. \end{array}$$

### 4.4. Value Function

The optimal decision on actions is stimulated by enhancing the value function defined as the maximum of the total discounted expected transmission rate from the current slot. In time slot *t*, when the remaining energy $E_{rem}\left(t\right)$ and belief of the primary channel $\mu \left(t\right)$ are given, based on the foregoing analysis, the value function, denoted as $\Upsilon \left(E_{rem}\left(t\right),\mu \left(t\right)\right)$, can be expressed as follows [37]:

(40) $$\begin{array}{c} \Upsilon \left(E_{rem}\left(t\right),\mu \left(t\right)\right)=\max_{a\left(k\right)\in A}\left\{\sum_{k=t}^{\infty }\delta^{k-t}\sum_{\Theta_{i}\in a\left(k\right)}\mathrm{Pr}\left(\Theta_{i}\right)\right.\times \sum_{E_{rem}\left(k+1\right)}\mathrm{Pr}\left[E_{rem}\left(k\right)\to E_{rem}\left(k+1\right)\left|\Theta_{i}\right.\right]\times \\ \left.R_{W}\left(E_{rem}\left(k\right),\mu \left(k\right),a\left(k\right)\left|\Theta_{i}\right.\right)\left|{}_{E_{rem}\left(k\right)=E_{rem}\left(t\right),\mu \left(k\right)=\mu \left(t\right)}\right.\right\} \end{array}$$

where δ:0<δ<1 denotes the discount factor, which indicates that the value of the reward in the current time slot is more than that of the next time slot. The optimal decision policy problem shown in Equation (40) can be solved by using the value iteration method given by Bertsekas [37].

### 4.5. Final Decision

In the current time slot, $t_{0}$, after the CSS phase, the decision for the operation of the CU and BS are detailed as follows. When the global decision indicates that the channel is occupied, both BS and CU trust this result and stay silent during the remaining of the current time slot. At the end of the time slot, belief $\mu \left(t_{0}+1\right)$ and remaining energy $E_{rem}\left(t_{0}+1\right)$ for use in the next time slot, $t_{0}+1$, are updated according to Equations (17) and (18), respectively; additionally, the transition probability of energy, $\mathrm{Pr}\left[E_{rem}\left(t_{0}\right)\to E_{rem}\left(t_{0}+1\right)\right]$, is given in Equation (19).

**Figure 5:**
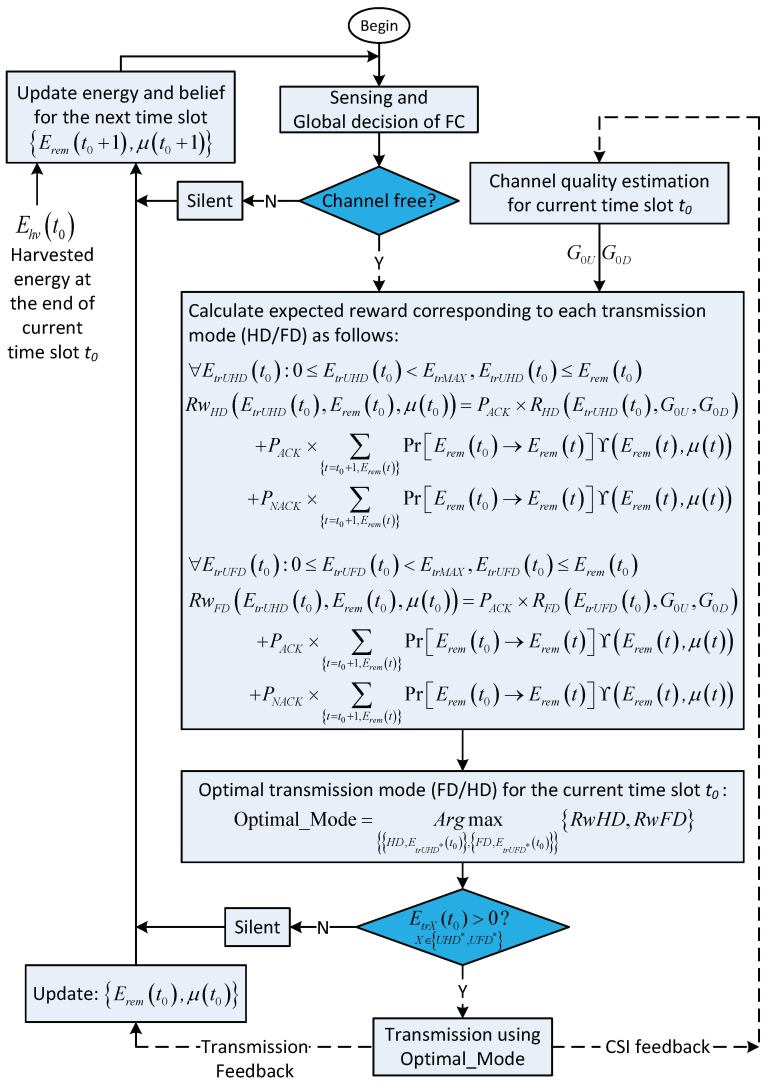
Flowchart for the operation of cognitive-user–base-station (CU–BS) transmission pair.

On the other hand, when the channel is vacant, the BS and CU decide the optimal transmission policy, which is either HD or FD transmission mode with the corresponding optimal transmission energy, $E_{trUHD}\left(t_{0}\right)$ and $E_{trUFD}\left(t_{0}\right)$, respectively, for maximizing the summation of the immediate reward in current time slot $t_{0}$ and the expected future reward from time slot $t=t_{0}+1$. The optimal decision policy depends on $\left\{E_{rem}\left(t_{0}\right),\mu \left(t_{0}\right),G_{0D},G_{0U}\right\}$, where $G_{0D}$ and $G_{0U}$ denote the channel gain on the downlink and uplink, respectively, in time slot $t_{0}$. We note that the values of $G_{0D}$ and $G_{0U}$ are assumed to be available. The formulas for immediate reward that correspond to HD or FD transmission mode can be denoted as $R_{HD}\left(E_{trUHD}\left(t_{0}\right),G_{0U},G_{0D}\right)$ and $R_{FD}\left(E_{trUFD}\left(t_{0}\right),G_{0U},G_{0D}\right)$, respectively. When the transmission is successful with probability $P_{ACK}=\mu \left(t_{0}\right)\left(1-p_{f}\right)$, $R_{HD}\left(E_{trUHD}\left(t_{0}\right),G_{0U},G_{0D}\right)$ and $R_{FD}\left(E_{trUFD}\left(t_{0}\right),G_{0U},G_{0D}\right)$ can be calculated with Equations (11) and (3), respectively; otherwise, when transmission is unsuccessful with probability $P_{NACK}=\left(1-\mu \left(t_{0}\right)\right)\left(1-p_{d}\right)$, zero reward is obtained. Based on the above analysis, the optimal decision polity for current time slot $t_{0}$ can be obtained as follows:

(41) PoEremt0,μt0,G0U,G0D=ArgmaxHD,EtrUHDt0,FD,EtrUFDt0PACK×RHDEtrUHDt0,G0U,G0D+PACK×∑t=t0+1,EremtPr∗ΥEremt,μt+PNACK×∑t=t0+1,EremtPr∗ΥEremt,μt,PACK×RFDEtrUFDt0,G0U,G0D+PACK×∑t=t0+1,EremtPr∗ΥEremt,μt+PNACK×∑t=t0+1,EremtPr∗ΥEremt,μt

where $\mathrm{Pr}\left[*\right]$ stands for $\mathrm{Pr}\left[E_{rem}\left(t_{0}\right)\to E_{rem}\left(t\right)\right]$, which is calculated according to Equation (19) and $\Upsilon \left(E_{rem}\left(t\right),\mu \left(t\right)\right)$ is calculated according to Equation (40). We note that, in Equation (41), $E_{rem}\left(t\right)$ and $\mu \left(t\right)$ are simplified notations that are calculated according to their observation context. Furthermore, the context consists of HD or FD transmission mode with the corresponding transmission energy, $E_{trUHD}\left(t_{0}\right)$ and $E_{trUFD}\left(t_{0}\right)$, respectively, and possible feedback (i.e., ACK , NACK) from the transmission at the end of time slot $t_{0}$. To summarize the scheduling of the CU–BS transmission pair, we show the flowchart of its operation for the entirety of its operation time in Figure 5.

## 5. The Actor–Critic Learning-Based Algorithm

In Section 4, the optimal decision policy is obtained by solving Equation (41), in which the expected future reward from the next time slot *t*, or the value function, $\Upsilon \left(E_{rem}\left(t\right),\mu \left(t\right)\right)$, is calculated by adopting the POMDP framework to solve Equation (40). Generally, the POMDP method requires a large number of formulation and computation to obtain the optimal policy. In this section, we formulate and solve Equation (41) based on the actor–critic learning method [38,39]. Although the actor–critic learning process may converge to a locally optimal policy [40], this method generates actions directly from the training policy; hence, it requires much less formulation and computation to obtain optimal actions compared to the POMDP framework.

**Figure 6:**
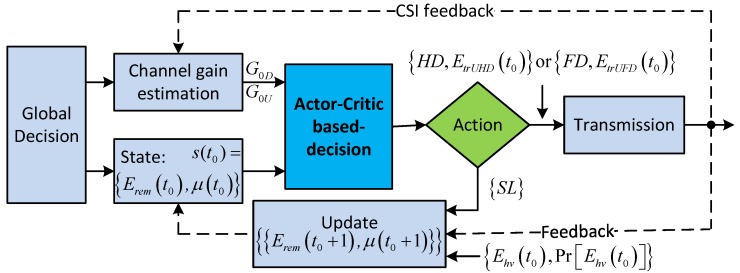
Flowchart of the proposed scheme when applying the actor–critic learning method.

To solve Equation (41), we need to find the value function, $\Upsilon \left(E_{rem}\left(t\right),\mu \left(t\right)\right)$, which is corresponding to each state $s\left(t\right)=\left\{E_{rem}\left(t\right),\mu \left(t\right)\right\}$. Now, we find the value function $\Upsilon \left(s\left(t\right)\right)$ based on the actor–critic learning method. The flowchart of this process is illustrated in Figure 6. In time slot $t=t_{0}+1$, given state s(t)∈S, each action a(t)∈A is accompanied by a corresponding immediate reward, $R_{W}\left(s\left(t\right),a\left(t\right)\right)$. Let $\Upsilon \left(s\left(t\right)\right)$ be the total discount reward of state s(t) corresponding to policy $\pi \left(t\right)=\left\{s(t),a(t)\right\}$. $\Upsilon \left(s\left(t\right)\right)$ can be given as [41]

(42) $$\Upsilon \left(s\left(t\right)\right)=E\left[\sum_{k=t}^{\infty }\gamma^{k-t}R_{W}\left(s\left(t\right),a\left(t\right)\right)\right]$$

where 0≤γ≤1 is the discount factor. We aim to find the optimal policy $\pi \left(t\right)$ that maximizes value function $\Upsilon \left(s\left(t\right)\right)$. Each action is selected according to a stochastic policy whose form follows a soft-max distribution (i.e., Gibbs or Boltzmann distribution) [38]. Let $h\left(s\left(t\right),a\left(t\right)\right)$ be the tendency to select action a(t) at state s(t). Policy function distribution is defined according to the Gibbs soft-max method as [38]

(43) $$\pi \left(a\left(t\right)|s\left(t\right)\right)=\mathrm{Pr}\left[a\left(t\right)\in A|s\left(t\right)\right]=\frac{e^{h\left(a\left(t\right),s\left(t\right)\right)}}{\sum_{a\in A}e^{h\left(a,s\left(t\right)\right)}}.$$

**Figure 7:**
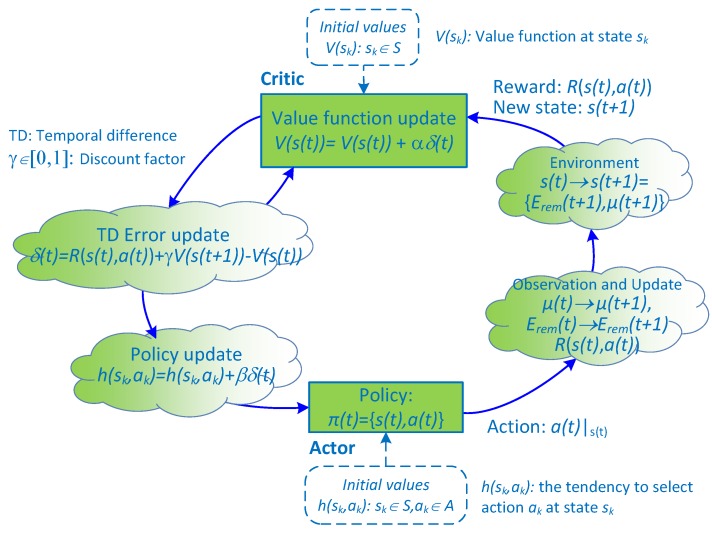
The actor–critic learning process of the proposed scheme.

Figure 7 shows the overall actor–critic learning process for finding the optimal policy for maximizing value function $\Upsilon \left(s\left(t\right)\right){|}_{s(t)\in S}$. The training process is detailed as follows. At the beginning of the time slot, the actor selects an action a(t)∈A with probability $\pi \left(a\left(t\right)|s\left(t\right)\right)$ when the system is at state s(t)∈S and starts the transmission process. At the end of the transmission, the system will determine the next state s(t+1) based on the amount of harvested energy and the amount of consumed energy during the time slot *t* and CSI according to the transmission feedback. When the action is silent or the transmission is unsuccessful, immediate reward $R_{W}\left(s\left(t\right),a\left(t\right)\right)$ will be zero; otherwise, $R_{W}\left(s\left(t\right),a\left(t\right)\right)$ is calculated with Equation (15) when the HD protocol is used and with Equation (8) when FD is used. We note that, when the amount of harvested energy during a time slot becomes greater than the storable space of the battery, the battery will overflow and the residual harvested energy will be wasted. To avoid entering this event, immediate reward $R_{W}\left(s\left(t\right),a\left(t\right)\right)$ will be set to zero if the battery overflows at the end of the time slot and the transmission energy corresponding to the selected action is not at the maximum level. Afterward, the temporal difference error is computed as $\delta \left(t\right)=\left[R_{W}\left(s\left(t\right),a\left(t\right)\right)+\gamma \Upsilon \left(s\left(t+1\right)\right)\right]-\Upsilon \left(s\left(t\right)\right)$, where δ(t) denotes the value of Υ(s(t)) before the action, as opposed to the value after observation. The value function is then updated as $\Upsilon \left(s\left(t\right)\right)=\Upsilon \left(s\left(t\right)\right)+\alpha \delta \left(t\right)$, and the tendency to select action is updated as $h\left(s\left(t\right),a\left(t\right)\right)=h\left(s\left(t\right),a\left(t\right)\right)+\eta \delta \left(t\right)$, where α and η are positive step-size parameters. Finally, policy π will be updated for use in the next state s(t+1). The training process terminates when a convergence is made. After the training phase, we can obtain the following output: the set of policy π and the set of value function $\Upsilon \left(s\left(t\right)\right)$ corresponding to each state s(t)∈S.

Up to now, we have adopted the actor–critic method to find the expected future reward, $\Upsilon \left(s\left(t\right)\right)$. Unlike the POMDP method, which requires numerous formulations and high implementation complexity to attain the optimal policy as described in Section 4, the actor–critic method allows us to obtain the optimal policy directly from the learning process without much formulation or implementation complexity. Compared to the POMDP method, the drawback of the actor–critic algorithm is that we cannot calculate the set of optimal policies corresponding to the set of system states in the form of off-line. Consequently, the actor–critic system needs a set of training data for its learning process. The set of training data should be sampled directly from the environment and should be large enough to fully characterize the variation of environment.

## 6. Evaluation

Performance of the proposed scheme, which is described in terms of average throughput, was measured through extensive simulation and compared with that of a conventional scheme [6,7,8]. In simulation, average throughput can be expressed as $\frac{1}{N}\sum_{i=1}^{N}R\left(i\right)$, where $R\left(i\right)$ is the immediate revenue obtained in time slot *i* and *N* is the total number of time slots used in simulation (*N* is 1000 time slots in our simulation). The difference in the operation of the conventional scheme compared to the proposed scheme can be described as follows. In a time slot, when the global decision indicates that the primary channel is vacant, since the conventional scheme only considers immediate reward, the CU and BS will greedily maximize throughput only for the current time slot by deciding on either the HD or the FD transmission mode using the highest amount of transmission energy. When operating in the context of energy harvesting, at the end of the time slot, the CU also updates information about its energy budget and the CSI of the channel for use in the next time slots.

In the simulation, the remaining energy $E_{rem}$ ranges between 0 and $E_{Bat}$ divided by 5; the values of belief μ are set as 0:0.05:1. The other simulation parameters are shown in Table 1. We note that, in the simulation, time slot duration is 200 ms and the rate of energy harvesting is about 15 mW [13]; hence, a packet of energy is equivalent to 167 μJ.

**Figure 8:**
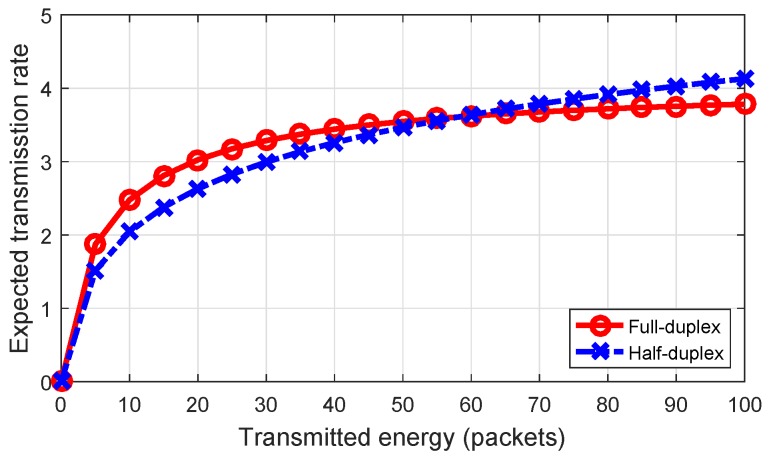
Expected transmission rate based on transmission energy (nats/s/Hz) in the CU when $G_{U}=G_{D}=-10$ (dB) and $\chi_{U}=\chi_{B}=\chi =0.01$.

Figure 8 compares the expected transmission rates of the HD link with that of the FD link calculated based on Equations (8) and (15), respectively. The figure shows that, when transmitted energy increases, the expected transmission rate of the FD link increases slowly due to the stronger effect of self-interference. Thus, based on the amount of transmitted energy, we can choose either the FD or the HD protocol to achieve a higher transmission rate.

### 6.1. The POMDP-Based Solution

**Figure 9:**
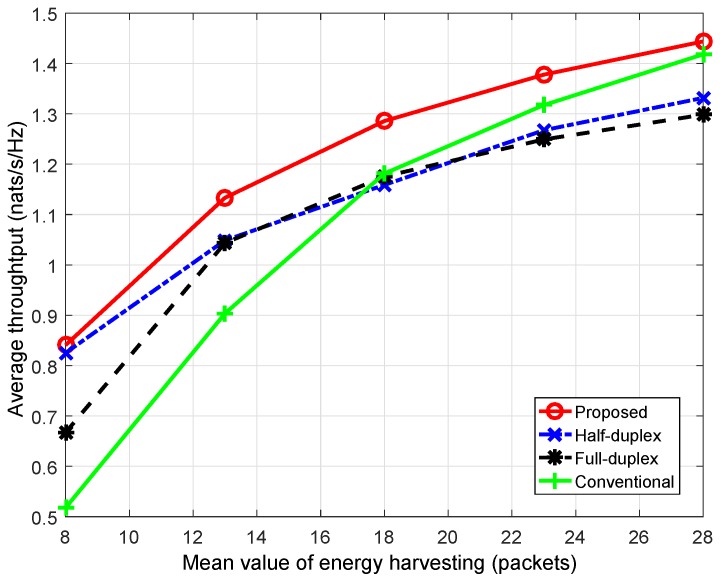
Average throughput according to the mean value of energy harvesting. The simulation was run for 1000 time slots, and the loop was executed 100 times.

In this section, Equation (40) is solved using the POMDP-based method. The optimal decision policy can be found after 19 iterations. First, we compare the performance under the proposed scheme with the conventional scheme in terms of average throughput. We note that the simulation condition is set to be the same for both schemes; in other words, the available energy budget (the amount of harvested energy) for use is the same. In addition, we also observed average throughput of the proposed scheme in the scenario where the CU can only use one transmission protocol (either HD or FD) i.e., the CU only allocates transmission energy. Figure 9 shows that average throughput from the proposed scheme greatly improves compared to that of the conventional scheme—remarkably when the energy harvesting rate is low. For instance, when the mean value of energy harvesting, $E_{hvmean}$, is 28, the proposed scheme gives 1.81% more throughput compared to the conventional scheme, whereas, when $E_{hvmean}$ is 8, the improvement is 62.37%. This can be explained based on the greedy decision of the conventional scheme. In a time slot, when the primary channel is vacant, the CU and BS in the conventional scheme will greedily use the highest amount of available energy for transmission as well as choose the transmission mode (either HD or FD), which provides a higher transmission rate to maximize immediate throughput only for the current time slot. This greedy algorithm may result in a lack of energy for use in the next time slots such that the CU has to stay in silent mode. Subsequently, for long-term operation, average throughput under the conventional scheme is generally decreased. On the other hand, since the proposed scheme aims to improve long-term throughput, the CU flexibly allocates the amount of transmitting energy among time slots as well as decides the corresponding transmission mode (either HD or FD) to maximize the summation of immediate throughput in the current time slot and the expected future reward resulting from the current decision. Therefore, the CU under the proposed scheme may possibly spend less power for the current transmission, or even stay silent to save energy for use in the next time slots. Obviously, it is probable that less immediate throughput is achieved in the current time slot, but more throughput will be gained in future.

In addition, we also observed the performance of the proposed scheme in two scenarios in which the CU can only operate in (i) HD mode and (ii) FD mode. It is known that the FD transmission can generally provide more throughput improvement compared to the HD mode. However, Figure 9 shows that the performance of FD mode is even worse than that of HD mode. This can be explained as follows. The performance of FD mode depends on not only the self-interference suppression technique and transmission power but also the channel gain between transmitter and receiver. In this work, when the channel gain on uplink and/or downlink becomes high, FD-link capacity greatly improves, whereas, when the channel gain worsens, the loss of the main links increases, which results in the domination of the interference link(s) at the receiver(s). This will seriously affect performance of the FD link; thus, in such cases, HD transmission will be better. Additionally, with awareness of these analyzed impacts, the idea of the HD/FD switching schedule in this work aims to take full advantage of both HD and FD transmission protocols.

**Figure 10:**
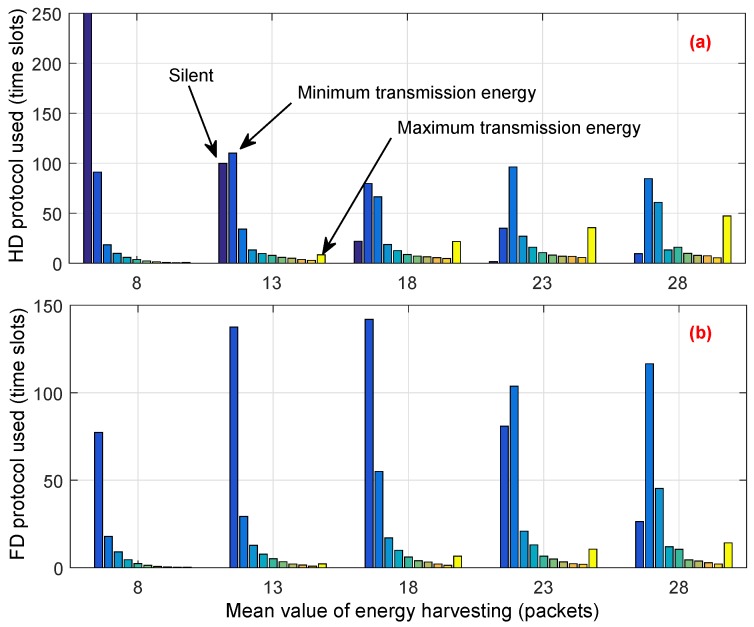
Statistics on the conventional scheme: average number of time slots where the CU decides to transmit using the (**a**) half-duplex (HD) or the (**b**) full-duplex (FD) protocol with the corresponding amounts of transmitted energy.

**Figure 11:**
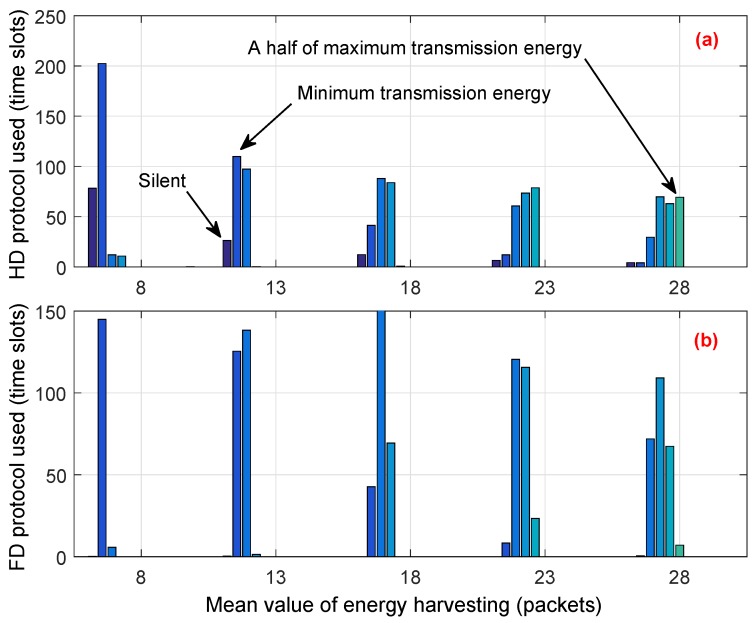
Statistics on the proposed scheme: average number of time slots where the CU decides to transmit using the (**a**) HD or the (**b**) FD protocol with the corresponding amounts of transmitted energy.

To explain how the proposed scheme can allocate an energy budget among time slots and utilize the vacant channel efficiently, in the simulation, we observed the decision of the CU for each time slot and counted the number of time slots where the CU transmitted by using either HD or FD transmission mode. The simulation was over 1000 time slots and the loop was 100 times. We note that, among 1000 time slots, the average number of vacant time slots is around 500. Simulation results are shown in Figure 10 and Figure 11. In both figures, each bar with the same color is aligned at the same position on the horizontal axis. The amount of transmission energy ranges between 0 and $E_{trMAX}$, and each group in the figures shows the distribution of transmitted energy from zero to the maximum value. Zero denotes that the CU proactively decides to stay in silent mode to save energy for use in future, or has to stay silent due to a lack of energy. In Figure 10a and Figure 11a, the dark-blue bar at the left of each group shows the total number of time slots where the CU has to stay or proactively stay in silent mode; the yellow bar shows the total number of slots where the CU decides to use maximal transmitted energy.

Comparing Figure 10a with Figure 11a, we see that the total number of time slots in which the CU in the conventional scheme stayed in silent mode is much higher than that of the proposed scheme. Moreover, the conventional scheme always decides to use the highest amount of transmission energy, which is reflected by the height of the yellow bars in Figure 10. This greedy approach results in the lack of energy budget for use in the next time slots. Therefore, the CU has to stay in silent mode or use a low level of energy for transmission in the next time slots. This is remarkably seen in Figure 10, where the distribution of transmission energy is mostly located on 10% to 20% (dark blue bars) of $E_{trMAX}$. On the other hand, the CU under the proposed scheme flexibly allocates the amount of transmission energy among time slots as well as in transmission mode. Moreover, the proposed scheme tries to prevent the CU from running out of energy in the subsequent time slots. Thus, the CU has more chances to stay active and transmit during its operation time, even when the energy harvesting rate is low. For example, when $E_{hvmean}$ is 8, Figure 11a shows that the average number of time slots where the CU has to stay silent is about 78, whereas, in the conventional scheme, the CU has to stay silent in about 250 time slots, as shown in Figure 10a. Moreover, Figure 11 shows that the proposed scheme avoids using the maximum transmitted energy, even when the energy harvesting rate is high. As an example, when $E_{hvmean}$ is 28, the distribution of the amount of transmitted energy is mostly located in the range between 30% and 50% of $E_{trMAX}$.

### 6.2. Actor–Critic-Based Solution

**Figure 12:**
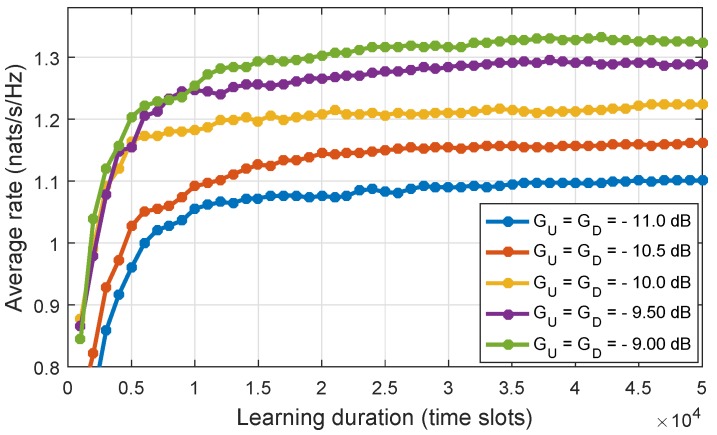
Convergence of the learning process for different mean values of channel gain.

Finally, we adopt the actor–critic method to solve Equation (41) where the set of value function $\Upsilon \left(.\right)$ can be obtained directly during the learning process. The training process is implemented as follows: during the learning process, we calculate the average rate after every batch of 1000 time slots, and we then calculate the different rate, ΔR, between two consecutive updates. We define the convergence condition as ΔR<ε. Generally, in the actor–critic algorithm, the training process often converge to a randomly local optimal policy. Therefore, in simulation, we repeat the learning process a number of times and select the policy that provides the maximum average rate. Figure 12 shows the average rate for different mean values of channel gain of the uplink and downlink when the learning process was executed on 50,000 time slots. When $\varepsilon =\frac{1}{2}10^{-3}$, the convergence condition can be matched after learning about 10,000–20,000 time slots.

**Figure 13:**
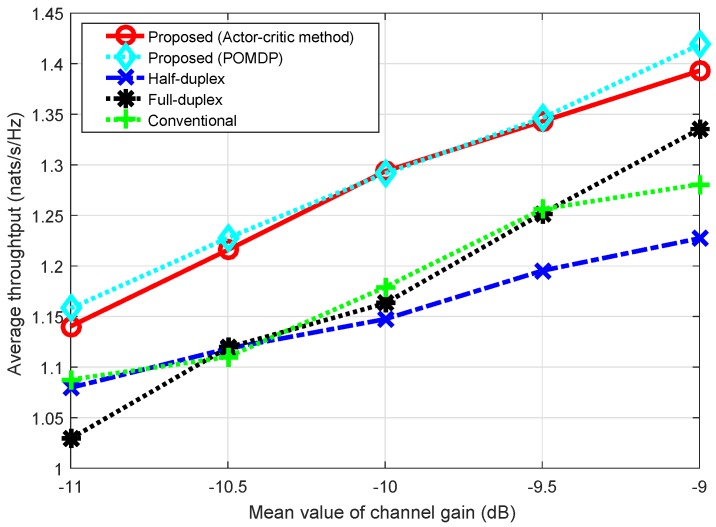
Average throughput according to the mean value of channel gain. The simulation was run for 1000 time slots, and the loop was executed 100 times.

Figure 13 shows the average throughput solved by the actor–critic algorithm compared with that of the POMDP method according to the mean value of channel gain when $G_{U}=G_{D}$. We can see that throughput by the POMDP method is slightly higher than that by the actor–critic method. Clearly, in the case of POMDP, the decision policy is obtained by searching the whole state space and action-space of the system; hence, optimal policy can be obtained. On the other hand, in the case of actor–critic algorithm, the policy is gradually reinforced during the learning process. Generally, the training process may converge to a randomly local optimal policy; thus, throughput by the actor–critic method can be slightly less than throughput by the POMDP method. As shown in the figure, the solution by the actor–critic method is nearly an optimal policy compared to the POMDP method. We note that the actor–critic method does not require a large number of formulations and computations to obtain the optimal policy.

In addition, we observed that, when the channel gain of the uplink and/or downlink is low, FD, compared to HD, gives less throughput due to the loss of the main links, resulting in the domination of the interference link(s) at the receiver(s); thus, in such cases, the HD transmission mode will be better. However, when the channel gain becomes high, received signals from the main links will be enhanced, which reduces the effect of the interference links, so the throughput of the FD protocol will improve. This work considers a switching schedule between the HD and FD transmission protocols in order to take full advantage of both the HD and FD protocols. Figure 13 shows that, when the channel gain of the uplink and downlink increases, there are greater chances that the FD transmission mode is selected; hence, the throughput of the proposed scheme greatly improves compared to that of the conventional scheme. For instance, when $G_{U}=G_{D}=-11$ dB, throughput improvement is 2.8% compared to that of the conventional scheme, whereas, when $G_{U}=G_{D}=-9$ dB, the improvement is 11.9%.

## 7. Conclusions

In this work, we jointly scheduled HD/FD transmission-mode switching and transmission-energy allocation in energy-harvesting-powered CRNs. In addition, we considered a practical scenario in which non-LOS fading channels, and the constraints in energy-harvesting capacity and battery capacity were also taken into account. Targeting long-term expected throughput, we presented two different solutions based on the POMDP framework and the actor–critic learning method, respectively. Our evaluation shows the advantages achieved by the proposed hybrid HD/FD scheme over the conventional scheme and the HD and FD schemes severally. In terms of the low energy-harvesting rate and/or low quality of non-LOS channel between the BS and the CU, the proposed scheme substantially outperformed the conventional scheme. In addition, the pros and cons of both solutions are also discussed.

We have mainly focused on improving the performance of each CU–BS transmission pair, which is assumed to be assigned to one specific primary channel throughout its transmission duration. Generally, for a general model of an entire energy-harvesting-powered CRN, it is worth considering multiple CUs utilizing multiple potential primary channels. In such a scenario, an interesting question is how CU–BS pairs can be effectively assigned to primary channels so as to further improve overall system performance. This is still an open issue that needs to be properly investigated in future works.

## Figures and Tables

**Table 1:** Simulation Parameters.

Symbol	Description	Initial Value
$G_{U}$	Uplink channel gain (mean value)	-10 dB
$G_{D}$	Downlink channel gain (mean value)	-10 dB
$\chi_{U},\chi_{B}$	Self-interference factor	0.01
$E_{Bat}$	Battery capacity	110 packets
$E_{trMAX}$	Maximum transmission energy	$E_{Bat}-10$
$E_{rem}$	Initial value of remaining energy	65 packets
$E_{trUHD}$	Transmission energy (HD mode)	$0:10:E_{trMAX}$
$E_{trUFD}$	Transmission energy (FD mode)	$0:10:E_{trMAX}$
$E_{SS}$	Spectrum sensing per time slot	3 packets
$E_{recFD}$	Receiving energy in FD mode	4 packets
$p_{d}$	Global probability of detection	0.9
$p_{f}$	Global probability of false alarm	0.1
μ	Belief for the primary channel	0.5
$p_{OV},p_{VO}$	State transition probability of the	0.2
	primary channel (see Figure 2)	
*T*	Duration of a time slot	200ms
$\tau_{SS}$	Duration of sensing and reporting	T/100
$\tau_{ACK}$	Duration of acknowledgement	T/200
δ	Discount factor in Equation (40)	0.95
α,β	Learning step-size parameters	0.4,0.3
